# Prevalence and Risk Factors for Sexually Transmitted Infections in Gay and Bisexual Prostate Cancer Survivors: Results From the *Restore-2* Study

**DOI:** 10.3389/fonc.2022.832508

**Published:** 2022-05-05

**Authors:** Christopher W. Wheldon, Elizabeth Polter, B. R. Simon Rosser, Alex J. Bates, Ryan Haggart, Morgan Wright, Darryl Mitteldorf, Michael W. Ross, Badrinath R. Konety, Nidhi Kohli, Kristine M. C. Talley, William West, Alexander K. Tatum

**Affiliations:** ^1^ Cancer Health Equity, Education and Research Lab, Department of Social and Behavioral Sciences, College of Public Health, Temple University, Philadelphia, PA, United States; ^2^ Division of Epidemiology and Community Health, School of Public Health, University of Minnesota, Minneapolis, MN, United States; ^3^ Department of Urology, University of Minnesota, Minneapolis, MN, United States; ^4^ Malecare Cancer Support, New York, NY, United States; ^5^ Department of Family Medicine and Community Health, University of Minnesota Medical School, Minneapolis, MN, United States; ^6^ Department of Urology, Rush Medical College, Chicago, IL, United States; ^7^ Department of Educational Psychology, University of Minnesota, Minneapolis, MN, United States; ^8^ Adult and Geriatric Health, University of Minnesota School of Nursing, Minneapolis, MN, United States; ^9^ Department of Writing Studies, University of Minnesota, Minneapolis, MN, United States; ^10^ Department of Counseling Psychology, Social Psychology, and Counseling, College of Health, Ball State University, Muncie, IN, United States

**Keywords:** oncology, STD (sexually transmitted disease), sexuality, homosexuality, risky health behaviors

## Abstract

**Background:**

Equitable cancer survivorship care for gay and bisexual male (GBM) prostate cancer survivors should be responsive to their sexual health needs. Rates of sexually transmitted infections (STIs) are higher among GBM compared to heterosexual men across the lifespan. In addition, evidence suggests that GBM will use a variety of strategies to cope with sexual dysfunction that may increase risk for STIs. The purpose of this study was to determine the prevalence of STIs following prostate cancer treatment among GBM and identify risk factors.

**Methods:**

In 2019, 401 GBM previously treated for prostate cancer were recruited into the Restore-2 Study. They completed a baseline online questionnaire with items assessing STIs diagnosed since being treated for prostate cancer. Any STI diagnoses was regressed on demographic, clinical, and relationship related variables using binary logistic regression.

**Results:**

Forty-five participants (11.4%) were diagnosed with an STI during or following their prostate cancer treatment. The mostly commonly diagnosed STI was syphilis (4.3%), followed by gonorrhoea (2.8%), and chlamydia (2.5%). Four participants were infected with HIV following their prostate cancer treatment. Independent risk factors for STI diagnosis included time since prostate cancer diagnosis (aOR = 1.18; 95% CI: 1.10-1.26), nonmonogamous sexual relationship (aOR = 11.23; 95% CI: 2.11-59.73), better sexual function (aOR = 1.02; 95% CI: 1.01-1.04), penile injection treatment (aOR = 3.28; 95% CI: 1.48-7.29), and multiple sex partners (aOR = 5.57; 95% CI: 1.64-18.96).

**Conclusions:**

GBM prostate cancer survivors are at risk for STIs. Culturally responsive STI prevention should be incorporated into cancer survivorship plans, particularly as men are treated for and regain sexual function over time.

## Introduction

Equitable cancer care for gay and bisexual men (GBM) with prostate cancer should be responsive to their specific sexual health needs. Prostate cancer treatments are known to adversely impact sexual functioning among men in the general population as well as among GBM (1–3). Psychosocial interventions to treat sexual dysfunction following treatment are largely designed for prostate cancer survivors who are in monogamous heterosexual relationships (4, 5). Thus, these approaches are not responsive to the experiences, specific needs, or health risks disproportionately affecting GBM. As a population, older GBM differ from older heterosexual men in ways that may have a significant impact on their sexual rehabilitation needs.

Anal intercourse is a common and culturally important sexual behavior for GBM across the lifecourse (6). Older GBM are also more likely to be single or in consensually nonmonogamous relationships compared to their heterosexual peers (7, 8). Some evidence suggests that GBM will use a variety of strategies to cope with sexual dysfunction following prostate cancer treatment, including changing roles in sex (e.g., from the insertive to receptive anal sex) and opening up previously monogamous relationships to other partners (5). Both coping strategies can increase risk for STIs. In addition, late and long-term effects of prostate cancer treatment, such as chronic radiation proctitis and inflammation, may increase the risk of sexually transmitted infections (STIs) for GBM engaging in receptive anal sex (9). Rates of STIs are higher among GBM at all ages compared to heterosexual men (10, 11); however, the prevalence and predictors of STIs among older GBM cancer survivors who have experienced sexual dysfunction has not been previously studied.

The purpose of this study was to determine the prevalence of STIs following prostate cancer treatment among GBM and to identify risk factors. We hypothesized that the following variables would be positively associated with a post-prostate cancer treatment STI diagnosis: (1) nonmonogamy in the primary relationship; (2) change to an anal receptive sex role; and (3) radiation treatment compared to surgery alone. We also explored the association of sociodemographic, clinical, and behavioral variables with STI diagnosis.

## Methods

Participants were enrolled in a clinical trial designed to test the effectiveness of an online rehabilitation program tailored for GBM prostate cancer patients and survivors (Restore-2). Inclusion criteria included (1) identifying as gay, bisexual, or reporting sex with a man, (2) having been previously or currently treated for prostate cancer (e.g., prostatectomy or radiation), and at study entry, having a significant sexual function and/or urinary function challenge resulting from treatment. Since the study website and all materials were online, Internet-using and ability to read English were implicit criteria. Participants were excluded if they could not read or write English or lived outside the United States or its territories. Inclusion and exclusion criteria were the same for the parent study. The total sample in the study was 401 men, although six participants were excluded from the current analysis due to missing data on key variables. Participants were recruited in urology clinics, print advertisements, and online through cancer support groups, dating applications, and social media sites. All participants completed an online eligibility screener and a vetting telephone call with a study staff member. All study assessments were online and self-reported. This study reports on baseline data collected in 2019. The full methodology for the Restore-2 study can be found elsewhere (12).

## Measures

### Sexually Transmitted Infections

Participants were asked to indicate if they ever had the following sexually transmitted infections “diagnosed before prostate cancer treatment” or “diagnosed after prostate cancer treatment”: HIV (or AIDS), syphilis, gonorrhea, chlamydia, HPV (genital or anal warts), herpes simplex virus (HSV), hepatitis A, hepatitis B, or hepatitis C. They could answer *yes* separately for each time point (i.e., before or after prostate cancer treatment). One participant who selected that they were diagnosed with HIV (N=1) both before and after prostate cancer were counted among participants diagnosed with HIV before, but not after prostate cancer. Participants who reported that they were diagnosed with syphilis (N=2), chlamydia (N=2), or HPV (N=1) both before and after cancer were counted in both categories. For the remaining STIs, no participant listed a diagnosis both before and after prostate cancer. Two composite variables were created to indicate any of the nine STIs before or after prostate cancer treatment, respectively.

### Sociodemographic Variables

Demographic questions were adapted from the U.S. Census and from the 2018 American Community Survey and included age, race/ethnicity, U.S. region, and educational attainment.

### Clinical Variables

Prostate cancer treatment type was investigated by asking participants to check which treatments they had undergone in nine categories, which at analysis were collapsed into: surgery or cryotherapy only, radiation only, hormone therapy (in combination with surgery and/or radiation), and other. Time since prostate cancer diagnosis was calculated in years.

### Relationship Characteristics

Current relationship status (single, dating, married or in a long-term relationship, or widowed, divorced no longer in a relationship) was assessed with two items. The first asked What is your current relationship status? Responses were combined into a dichotomous variable to contrast participants who were married or in a long-term relationship with those who were single, dating, widowed, divorced or no longer in a relationship. If the response was married or in a long-term relationship with a man, a follow-up question (How many years have you been in a relationship with this man)? was used to document years in current relationship with a man. Five participants who were in a relationship with a woman were not asked this follow-up question. A participant was considered to be in a non-monogamous relationship if they were married, or in a long-term relationship and reported either (1) one sexual partner who was not their primary partner in the past month or (2) two or more sexual partners in the past month.

### Disease Specific Quality of Life and Sexual Function

The Expanded Prostate Cancer Index Composite (EPIC-50) measures for the frequency and perceived bother of bowel and sexual symptoms were included. The subscale reliability (r≥0.80) and internal consistency were adequate (α≥ 0.82) (13). All scales total 100, with higher scores indicating better functioning or less bother. To assess treatments used for sexual function, participants were provided with a list of treatment options (e.g., *Viagra, Cialis, or other erectile enhancing drugs*; *vacuum pump to help with erections*; and *Penile injections (e.g., Coverjet)*) and asked which of the following they tried in the last 90 days. They could select more than one treatment. For this analysis, they were classified as having used the treatment if they reported using it at least once in the previous 90 days.

### Behavioral Risk Factors

Risky alcohol use was measured with two items that asked (1) how often a participant had a drink containing alcohol (e.g., a 12 ounce can or glass of beer or cooler, a 5-ounce glass of wine, or a drink containing 1 shot of liquor and (2) how many alcoholic drinks a participant had on a typical day when drinking alcohol. Participants were classified as having risky alcohol use if they indicated more than five drinks on any day in the previous year or more than 5 drinks per day on a typical day when drinking (14). Change in sex role was assessed in an item asking how often they had changed their sex role (from “top” to “bottom”) to help with the sexual effects of prostate cancer treatment in the past ninety days. Participants were classified as changing their role in sex if they reported a change in role at least once in the previous 90 days. Participants were asked “in the last three months, with how many men (including your partner or spouse, if applicable) have you engaged in any kind of sex? (0, 1, or more than 1).

### Data Analysis

Frequencies and percentages were calculated for the prevalence of each STI after prostate cancer treatment. Variables including demographics, prostate cancer treatment and time since diagnosis, relationship variables, disease-specific quality-of-life, use of therapies for erectile function, and behavioral risk variables, were calculated for participants with and without a post-prostate cancer STI diagnosis using means and standard deviations (for continuous variables) and frequencies and percentages (for categorical variables). Logistic regression was used to calculate the association of post-prostate cancer STI with each characteristic. A multivariable model was used to identify the strongest independent correlates of STI diagnosis. All hypothesis tests were two-sided, with significance level of p ≤ 0.05. Sample size estimates were based on the parent study (12). All analyses were conducted in Stata version 16 (StataCorp. 2019. *Stata Statistical Software: Release 16*. College Station, TX: StataCorp LLC).

## Results

The characteristics of the sample are described in **Table 1**. The sample was primarily non-Hispanic White (87.6%) with an average age of 63.5 (SD=6.7). Prostate cancer treatment was primarily surgery (58.2%) with an average of 5.3 (SD=4.9) years since diagnosis. Most (62.1%) had used an erectile enhancing drug to treat sexual dysfunction. Approximately half were married or in a long-term relationship with a man (49.0%) and most reported a lifetime STI prior to their cancer diagnosis (55.7%).

**Table 1:** Correlates of acquiring sexually transmitted infection (STI) post prostate cancer treatment among gay and bisexual men (N = 395).

				Bivariate Models		Adjusted Models^b^	
	Entire Sample (N = 395)	No STI (N = 350)	STI (N = 45)	OR (95% CI)	p-value	aOR (95% CI)	p-value
	n (%)^a^	n (%)	n (%)				
**Sociodemographic variables**							
Age, mean (SD)	63.5 (6.7)	63.5 (6.6)	63.2 (6.7)	0.99 (0.95-1.04)	0.74	0.97 (0.91-1.02)	0.25
Race/Ethnicity							
White and non-Hispanic	346 (87.6%)	305 (87.1%)	41 (91.1%)	1.00			
Non-white or Hispanic	49 (12.4%)	45 (12.9%)	4 (8.9%)	0.66 (0.23-1.93)	0.45	0.65 (0.20-2.11)	0.48
Region							
United States West	107 (27.1%)	94 (26.9%)	13 (28.9%)	1.00		1.00	
United States South	127 (32.2%)	109 (31.1%)	18 (40.0%)	1.19 (0.56-2.57)	0.65	1.81 (0.76-4.31)	0.18
United States Midwest	74 (18.7%)	70 (20.0%)	4 (8.9%)	0.41 (0.13-1.32)	0.14	0.73 (0.21-2.56)	0.63
United States Northeast	87 (22.0%)	77 (22.0%)	10 (22.2%)	0.94 (0.39-2.26)	0.88	1.24 (0.46-3.32)	0.67
Education							
High School, GED, associate’s degree or some college	92 (23.3%)	83 (23.7%)	9 (20.0%)	1.00		1.00	
Bachelor’s degree	131 (33.2%)	111 (31.7%)	20 (44.4%)	1.66 (0.72-3.84)	0.23	1.99 (0.78-5.07)	0.15
Graduate degree	172 (43.5%)	156 (44.6%)	16 (35.6%)	0.95 (0.40-2.23)	0.90	1.07 (0.41-2.78)	0.88
**Clinical variables**							
Treatment type							
Surgery alone	230 (58.2%)	203 (58.0%)	27 (60.0%)	1.00		1.00	
Radiation alone (external or brachytherapy)	74 (18.7%)	64 (18.3%)	10 (22.2%)	1.17 (0.54-2.56)	0.69	1.39 (0.57-3.38)	0.47
Hormone Therapy	65 (16.5%)	61 (17.4%)	4 (8.9%)	0.49 (0.16-1.46)	0.20	1.01 (0.31-3.36)	0.98
Other	26 (6.6%)	22 (6.3%)	4 (8.9%)	1.37 (0.44-4.27)	0.59	1.29 (0.36-4.57)	0.70
Time since diagnosis (median years, interquartile Range)	4.0 (2.0-8.0)	3.0 (2.0-7.0)	8.0 (5.0-12.0)	**1.12 (1.06-1.18)**	**<0.001**	**1.18 (1.10-1.26)** ^c^	**<0.001**
**Relationship variables**							
Relationship Status							
Married/in a long-term relationship	193 (49.0%)	174 (49.9%)	27 (60.0%)	1.00		1.00	
Single, dating, widowed, divorced, no longer in a relationship	201 (51.0%)	175 (50.1%)	18 (40.0%)	1.51 (0.80-2.84)	0.20	1.41 (0.70-2.82)^d^	0.33
							
Years in current relationship^d^							
0-<5 years	35 (18.6%)	33 (19.4%)	2 (11.1%)	1.00		1.00	
5 or more years	153 (81.4%)	137 (80.6%)	16 (88.9%)	1.93 (0.42-8.80)	0.39	2.42 (0.43-13.59)	0.31
Nonmonogamous sexual relationship ^e^	87 (45.5%)	71 (41.0%)	16 (88.9%)	11.49 (2.56-51.55)	0.001	11.23 (2.11-59.73)^f^	0.004
**Disease specific quality of life and sexual function**							
EPIC Sexual Function	35.5 (21.2)	34.6 (21.4)	42.9 (18.5)	1.02 (1.00-1.03)	<0.001	1.02 (1.01-1.04)^g^	0.01
EPIC Sexual Bother	39.2 (26.3)	38.9 (26.8)	41.8 (22.3)	1.00 (0.99-1.02)	0.48	0.99 (0.97-1.01)	0.35
EPIC Bowel Function	76.9 (9.4)	77.0 (9.3)	76.3 (10.2)	0.99 (0.96-1.02)	0.62	0.97 (0.94-1.01)	0.18
EPIC Bowel Bother	85.7 (15.5)	85.8 (15.1)	85.0 (18.4)	1.00 (0.98-1.02)	0.76	0.99 (0.97-1.01)	0.38
Use of therapies for erectile dysfunction (reference category is no/not selected for each treatment)							
Oral medication	244 (62.1%)	218 (62.6%)	26 (57.8%)	0.82 (0.43-1.53)	0.53	0.50 (0.23-1.07)	0.07
Pump	85 (21.6%)	77 (22.1%)	8 (17.8%)	0.76 (0.34-1.71)	0.57	0.61 (0.24-1.56)	0.30
Injections	73 (18.5%)	58 (16.6%)	15 (33.3%)	2.51 (1.27-4.96)	0.007	3.28 (1.48-7.29)^h^	0.004
**Behavioral risk variables**							
Risky drinking: >5 drinks on any day in the past year OR >5 drinks per day on average	97 (29.7%)	85 (29.5%)	12 (30.8%)	1.06 (0.51-2.19)	0.87	0.88 (0.38-2.01)	0.76
STI prior to PCa Dx	220 (55.7%)	197 (56.3%)	23 (51.1%)	0.81 (0.44-1.51)	0.51	0.79 (0.39-1.60)	0.51
Change in sex role	92 (23.5%)	79 (22.8%)	13 (28.9%)	1.37 (0.69-2.74)	0.37	0.68 (0.30-1.53)	0.35
Number of total sex partners include oral and anal							
0	98 (24.9%)	94 (26.9%)	4 (8.9%)	1.00		1.00	
1	128 (32.5%)	120 (34.4%)	8 (17.8%)	1.57 (0.46-5.36)	0.47	1.51 (0.40-5.76)	0.46
2 or more	168 (42.6%)	135 (38.7%)	33 (73.3%)	5.74 (1.97-16.76)	0.001	5.57 (1.64-18.96)^i^	0.006

a Mean and standard deviation or median and interquartile range replace frequency and percent for continuous variables where noted.

b Adjusted for years since diagnosis, nonmonogamous relationship (if in a relationship), use of injections to treat ED, EPIC sexual function, and number of total sex partners unless otherwise noted.

c Adjusted for nonmonogamous relationship (if in a relationship), use of injections to treat ED, EPIC sexual function, and number of total sex partners.

d Adjusted for years since diagnosis, use of injections to treat ED, EPIC sexual function, and number of total sex partners.

e Among 188 men who reported a current relationship with a man.

f Adjusted for years since diagnosis, use of injections to treat ED, and EPIC sexual function.

g Adjusted for years since diagnosis, use of injections to treat ED, and number of total sex partners.

h Adjusted for years since diagnosis, nonmonogamous relationship (if in a relationship), EPIC sexual function, and number of total sex partners.

i Adjusted for years since diagnosis, use of injections to treat ED, and EPIC sexual function.

Bolded values indicate p < 0.05.

Forty-five participants (11.4%) answered that they had been diagnosed with an STI following their prostate cancer treatment (**Table 2**). The mostly commonly diagnosed STI was syphilis (4.3%), followed by gonorrhoea (2.8%), and chlamydia (2.5%). Four participants (1.0%) were infected with HIV following their prostate cancer treatment.

**Table 2:** Frequency of sexually transmitted infections among gay and bisexual male prostate cancer survivors since their prostate cancer treatment (N = 395).

Type of Sexually Transmitted Infection	N	%
Any	45	11.4
Syphilis	17	4.3
Gonorrhea	11	2.8
Chlamydia	10	2.5
Human papillomavirus (Genital or Anal Warts)	9	2.3
HIV	4	1
Herpes Simplex Virus (HSV)	4	1
Hepatitis C	4	1
Hepatitis B	3	0.8
Hepatitis A	2	0.5

Independent risk factors for STI diagnosis (**Table 1**) included time since prostate cancer diagnosis (aOR = 1.18; 95% CI: 1.10-1.26), nonmonogamous sexual relationship (aOR = 11.23; 95% CI: 2.11-59.73), better sexual function (aOR = 1.02; 95% CI: 1.01-1.04), penile injection treatment (aOR = 3.28; 95% CI: 1.48-7.29), and multiple sex partners (aOR = 5.57; 95% CI: 1.64-18.96).

## Discussion

The acquisition of STIs among GBM prostate cancer survivors is an area that has received almost no attention in the clinical literature. STIs among adults older than age 50 are relatively uncommon in the general population, but GBM remain at risk of acquiring new infections into late adulthood (11, 15). Our findings confirm that GBM prostate cancer survivors with treatment-related sexual dysfunction are still at high-risk for STIs. Approximately 1 in 9 of the survivors in this study reported acquiring an STI following their prostate cancer treatment. The most commonly acquired STI was syphilis, which reflects established trends in syphilis incidence among GBM in the U.S (16). The prevalence of syphilis among men in the current study (4.3%) was higher than the point prevalence in a recent study of GBM in North American and Europe, but still within range of estimates(3.4%; 95% CI: 1.8% to 5.4%) (17).

Several behavioral and clinical correlates of STIs were identified in the current study. As hypothesized, for participants in long-term relationships, nonmonogamy was associated with increased risk of STIs. Nonmonogamous relationship agreements are common among GBM (8), particularly among older age cohorts and among those in long-term relationships of five or more years (18). Clinicians treating GBM for prostate cancer related sexual dysfunction should not make the assumption that patients who are married or with a long-term partner are monogamous (19). Taking a system-wide approach to sexual health in cancer survivorship care can help to overcome organizational and interpersonal barriers to patient-provider sexual health communication (20).

Another behavioral factor associated with STI risk was having multiple recent oral or anal sex partners; however, the hypothesis about role change in sex was not supported. In other words, we failed to find evidence that changing from an insertive (i.e., “top”) to receptive (i.e., “bottom”) role after prostate cancer treatment was associated with greater likelihood of STIs. Despite the null finding, nearly 1 in 4 participants described a change in role. These role changes may result from decreased erectile functioning following PCa treatment and highlight the importance of STI prevention in this population.

Radiation treatment was also not associated with STIs in this study. It has been hypothesized that inflammatory conditions like proctitis may increase susceptibility to STI acquisition through receptive anal sex (21). While we did not measure proctitis directly in this study, radiation proctitis is a potential long term effect of prostate cancer treatment (9). Proctitis can also result from untreated STIs (22), thus it is important for oncologists treating bowel dysfunction in GBM with prostate cancer to test for STIs. Future studies should also continue to examine the role of treatment related inflammatory bowel conditions on STI risk for those engaging in receptive anal sex.

With regard to other clinical factors, years since prostate cancer diagnosis was positively associated with STI diagnosis. This finding may reflect the timeframe used to assess STI diagnoses (i.e., since prostate cancer treatment) or time-based improvements in sexual function. Relatedly, better sexual function and use of penile injections for erectile dysfunction were both independently associated with STIs; however, other treatments for erectile dysfunction were not associated with STI risk. It is unclear why penile injections, and not oral medications (e.g., sildenafil), were associated with increases in STI acquisition. Future research should explore potential explanations such as higher efficacy of injections in producing erections rigid enough for anal intercourse, the context of use, and the potential that a wound at the injection site may increase risk for STI transmission.

It is important to note that lifetime history of STIs was not predictive of post-prostate cancer treatment STIs, suggesting that risk was not solely concentrated among men with a history of STIs. The process of sexual rehabilitation following treatment for prostate cancer may involve additional behavioral and psychosocial changes that increase STI risk. For example, men may forgo the use of condoms to help manage erectile dysfunction. Safer sex negotiations with existing and new partners may also be impacted by lowered sexual self-esteem resulting from long term sexual dysfunction. Terror management theory suggests that a mortality threat (e.g., cancer) can increase sexual risk behaviors among men (23). It is theorized that when faced with a mortality threat, men may cope by engaging in self-enhancing sexual behaviors. Further research is needed to directly test this hypothesis in cancer survivors.

There are several limitations to the research design. For many of the associations reported, the temporality relative to STI acquisition could not be established. Thus, the associations do not represent cause and effect relationships. Furthermore, the measures were self-reported with no objective verification of STI diagnoses possibly leading to underreporting. External validity is limited by the non-probability sample of mostly highly educated non-Hispanic white men. Finally, the estimated associations may be unreliable due to limited distributions in some variables resulting in wide confidence intervals. Replication of these findings on ethnoracially and socioeconomically diverse samples is essential.

Despite these limitations, this analysis has several strengths. We recently conducted a systematic review of all publications (from 1995 to 2022) on GBM prostate cancer patients in English, Spanish and French. Based on this review, we confirm this is the first study to examine the prevalence and risk factors for STIs among GBM prostate cancer survivors. The findings indicate that older GBM who have completed treatment for prostate cancer and have experienced significant sexual dysfunction are still at risk for acquiring new STIs. Cancer survivorship and sexual rehabilitation care plans for GBM following prostate cancer treatment should include STI prevention.

## Data Availability Statement

The raw data supporting the conclusions of this article will be made available by the authors, without undue reservation.

## Ethics Statement

The studies involving human participants were reviewed and approved by The Institutional Review Board at the University of Minnesota. The patients/participants provided their written informed consent to participate in this study.

## Author Contributions

Conceptualization, BR, KT, CW, DM, MR, BK, and NK; Methodology, BR, NK, EP, and MW; Validation, NK and EP; Formal Analysis, NK, EP, and MW; Investigation, BR, EP, MW, RH, and NK; Data Curation, RH, MW, and NK; Writing – Original Draft, BR, EP, and KT; Writing – review and editing, all authors; Supervision, BR and NK; Project administration, BR, BK, and NK; Funding acquisition, BR, KT, CW, WW, DM, MR, BK, and NK; All authors contributed to the article and approved the submitted version.

## Funding

This study is supported by funding from the National Cancer Institute (NCI) [1R01CA218657; PI: Rosser]. The content is solely the responsibility of the authors and does not necessarily represent the official views of the National Institutes of Health.

## Conflict of Interest

The authors declare that the research was conducted in the absence of any commercial or financial relationships that could be construed as a potential conflict of interest.

## Publisher’s Note

All claims expressed in this article are solely those of the authors and do not necessarily represent those of their affiliated organizations, or those of the publisher, the editors and the reviewers. Any product that may be evaluated in this article, or claim that may be made by its manufacturer, is not guaranteed or endorsed by the publisher.
